# A case of IgE myeloma transformed into IgE-producing plasma cell leukaemia

**DOI:** 10.11613/BM.2020.010801

**Published:** 2019-12-15

**Authors:** Nicolas Galakhoff, Cyril Leven, Jean-Richard Eveillard, Adrian Tempescul, Hélène Kerspern, Cécile Aubron, Caroline Buors, Éric Lippert, Jean-Luc Carré, Maël Padelli

**Affiliations:** 1 Department of Biochemistry and Pharmaco-Toxicology, Brest University Hospital, Brest, France; 2 Department of Haematology, Brest University Hospital, Brest, France; 3 Medical Intensive Care, Brest University Hospital, Brest, France; 4 Laboratory of Haematology, Brest University Hospital, Brest, France; 5 Université de Brest, INSERM, EFS, UMR 1078, GGB, Brest, France; 6 Department of Biochemistry and Pharmaco-Toxicology, Martinique University Hospital, Fort-de-France, France

**Keywords:** immunofixation, immunoglobulin E, monoclonal gammopathy of undetermined significance, multiple myeloma, plasma cell leukaemia

## Abstract

This is a case report of a challenging diagnosis of IgE monoclonal gammopathy of undetermined significance, which transformed into myeloma, then transformed into IgE-producing plasma cell leukaemia in a 71-year-old male who was followed in Brest, France, from 2015 to 2019. The IgE-producing variant is the rarest sub-type of multiple myeloma, and plasma cell leukaemia is considered to be the rarest and the most aggressive of human monoclonal gammopathies. In November 2015, hypogammaglobulinemia was detected during a systematic check-up. A kappa light chain monoclonal gammopathy was first diagnosed due to an increase of the free kappa/lambda light chains ratio. No monoclonal immunoglobulin was detected by either serum protein electrophoresis (Capillarys 2, Sebia, Issy-les-Moulineaux, France) or immunofixation (Hydrasys 2, Sebia, Issy-les-Moulineaux, France). In June 2018, a blood smear led to the diagnosis of plasma cell leukaemia. A monoclonal peak was detected and identified as IgE-kappa. Analysis of an archival sample taken three years earlier, revealed the presence of a monoclonal IgE, which had been missed at diagnosis. Chemotherapy with bortezomib and dexamethasone was introduced. The patient survived 10 months after the diagnosis of leukaemia. This case shows that an abnormal free light chain ratio should be considered as a possible marker of IgE monoclonal gammopathy even in the absence of a solitary light chain revealed by immunofixation. In addition, the use of an undiluted serum may increase the sensitivity of the immunofixation for the detection of IgE monoclonal gammopathies compared to the 1:3 dilution recommended by the manufacturer.

## Introduction

Multiple myeloma (MM) is a haematological malignancy characterized by the clonal proliferation of plasma cells in the bone marrow leading to overproduction of a monoclonal immunoglobulin (*1*). It constitutes 1% of all cancers and over 10% of all haematological malignancies (*2*). The plasma cells of monoclonal gammopathy of undetermined significance (MGUS) are immunoglobulin producing premalignant precursor tumours of MM that are stable and not associated with the presence of the secondary clinical manifestations including skeletal lytic lesions, anaemia, immunodeficiency, renal failure and hypercalcemia (*3*). They are derived from long-lived, post-germinal center B cells, which are activated B cells differentiated into plasma blasts that typically migrate back to the bone marrow where they become terminally differentiated long-lived plasma cells. Monoclonal gammopathy of undetermined significance is present in about 4% of Caucasians over the age of 50, with a 1% average annual risk of progression to malignant MM (*4*). The IgE-producing variant is the rarest sub-type of MM accounting for less than 0.1% of MM cases (*5*). About 60 cases have been described in the literature so far highlighting that IgE MM seems to have the poorest outcomes among heavy chain subtypes of MM with a mean survival time of 16 months *versus* 30 months for non IgE MM (*6*).

Plasma cell leukaemia (PCL) is considered to be the rarest of human monoclonal gammopathy, accounting for 0.04 cases *per* 100,000 persons *per* year in Europe and also the most aggressive (*7*). It can either develop *de novo* (primary PCL) or evolve as a late-stage complication of MM (secondary PCL, sPCL) occurring in less than 1% of MM cases (*8*). Plasma cell leukaemia is a serious MM complication defined by the presence of > 2000/mm^3^ plasma cells in the peripheral blood or > 20% of the total white blood cell count (*9*). The median overall survival of 1 to 2 months (*10*, *11*). Secondary PCL is characterized by a multistep accumulation of adverse biological features in patients with advanced relapsed and/or refractory MM (*12*). Unlike MM cells, PCL cells are poorly dependent on the bone marrow microenvironment for their growth and survival. The neoplastic plasma cells are more prone to enter the blood stream due to changes in expression of adhesion molecules, chemokine receptors, and the presence of molecular aberration promoting tumour growth outside the bone marrow, inhibition of apoptosis, and escape from immune surveillance (*13*).

The diagnosis of monoclonal gammopathy (MG) requires the laboratory’s great vigilance, especially when a heavy chain D or E is involved. The International Myeloma Working Group provides guidelines that recommend performing serum protein electrophoresis (SPE), serum quantitation of free light chains (FLC) and serum immunofixation electrophoresis (IFE) testing for IgA, IgG, IgM, kappa and lambda light chains in the screening of MG (*14*, *15*). When an isolated light chain monoclonal band is detected in IFE, IgD and IgE IFE must be performed to distinguish a light chain MG and an IgD or IgE MG. On the other hand, when the FLC quantitation detects an imbalance in light chain production no guidelines, to our knowledge, propose to perform IgD and IgE IFE.

The aim of this case report was to illustrate that an abnormal serum FLC result can reveal IgE monoclonal gammopathy even when no light chain monoclonal bands are detected in IFE. We report here a challenging diagnosis of IgE MGUS transformed into MM transformed into IgE-producing sPCL.

## Case report

**Figure 1:**
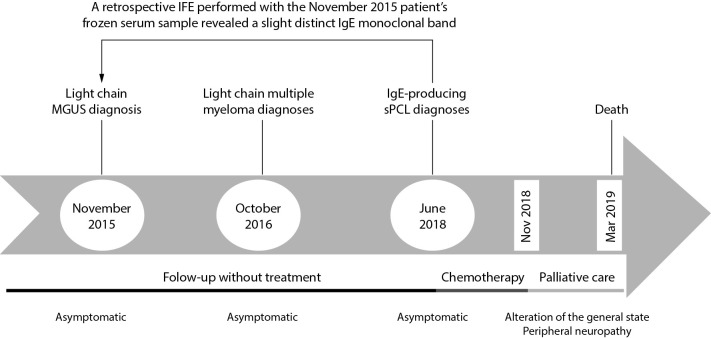
Time line summarizing the relevant patient data and diagnoses. IFE - immunofixation electrophoresis performed with Hydrasys 2. IgE - Immunoglobulin E; MGUS - Monoclonal gammopathy of undetermined significance; sPCL - Secondary plasma cell leukaemia.

In November 2015, a 71-year-old male was referred to the haematology department of Brest University Hospital (France) after the systematic discovery of hypogammaglobulinemia. A time line summarizing patient follow-up from 2015 to 2019 is presented in Figure 1. His past medical history was confined to high blood pressure, gastroesophageal reflux, inguinal hernia surgery, and an uninvestigated mild renal impairment. He was under antihypertension treatment by nebivolol and indapamide. At the time of haematological consultation, the patient was asymptomatic and the physical examination was unremarkable. Laboratory testing (Table 1), showed an elevated serum creatinine concentration of 131 µmol/L corresponding to a glomerular filtration rate of 47 mL/min estimated by the Chronic Kidney Disease Epidemiology Collaboration (CKD-EPI) equation and an elevated serum β2-microglobulin of 2.85 mg/L (*16*). There was no hypercalcaemia, no hyperproteinaemia and the blood count showed no particularity. The SPE performed by capillary electrophoresis with a Capillarys 2 instrument (Sebia, Issy-les-Moulineaux, France) showed important hypogammaglobulinemia of 4.8 g/L but no monoclonal component. Serum IFE performed in agarose gel with a Hydrasys 2 instrument (Sebia, Issy-les-Moulineaux, France) using IgG, IgA, IgM, kappa and lambda antisera provided no evidence of monoclonal protein band. The IgD and IgE antisera had not been tested due to the absence of monoclonal light chain in the IFE. Serum FLC quantification performed with a BN ProSpec instrument (Siemens, Erlangen, Germany) showed an excess of free kappa light chains (FKLC) of 163 mg/L, 6 mg/L of free lambda light chains (FLLC), and an increased FKLC/FLLC ratio of 27.2. The research of Bence-Jones proteinuria was not performed at that time. No bone lesion was observed by standard X-ray imaging and the disease was classified as light-chain MGUS. No treatment was deemed to be indicated at the time and a regular follow-up was scheduled.

**Table 1:** Laboratory results

**Parameter, unit**	**Results** **(November 2015)**	**Results** **(June 2018)**	**Results** **(November 2018)**	**Reference interval**
Red blood cells, x10^12^/L	4.73	3.09	3.03	4.50 – 6.50
Haemoglobin, g/L	130	101	106	130 – 170
Haematocrit, L/L	0.392	0.292	0.307	0.400 – 0.540
MCV, fL	82.9	94.5	101.3	82.0 – 98.0
MCH, pg	27.5	32.7	35	> 27.0
MCHC, g/L	332	346	345	315 – 365
Platelets, x10^9^/L	217	79	184	150 – 400
Leukocytes, x10^9^/L	7.5	80.3	3.1	4.0 – 10.0
Neutrophils, x10^9^/L	5.90	10.44	1.56	1.50 – 7.00
Lymphocytes, x10^9^/L	1.31	1.21	0.52	1.50 – 4.00
Monocytes, x10^9^/L	0.22	4.02	0.93	0.20 – 1.00
Eosinophils, x10^9^/L	0.00	0.80	0.03	0.00 – 0.50
Basophiles, x10^9^/L	0.04	0.00	0.05	0.00 – 0.20
Albumin, g/L	43.5	35.5	35.9	40.2 – 47.6
α1, g/L	2.7	5.3	4.7	2.1 – 3.5
α2, g/L	6.0	6.4	7.2	5.1 – 8.5
β1, g/L	4.0	2.9	3.1	3.4 – 5.2
β2, g/L	2.2	3.0	2.0	2.3 – 4.7
γ, g/L	4.8	4.6	1.9	8 – 13.5
Peak, g/L	absence	3.4	absence	NA
**Parameter, unit**	**Results** **(November 2015)**	**Results** **(June 2018)**	**Results** **(November 2018)**	**Reference interval**
Immunofixation	absence	Monoclonal IgE-Kappa	Monoclonal IgE-Kappa	NA
Immunoglobulin G, g/L	5.3	2.2	1.2	6.1 – 13.0
Immunoglobulin A, g/L	0.760	0.180	0.310	0.400 – 3.50
Immunoglobulin M, g/L	0.28	< 0.20	0.86	0.50 – 3.0
FKLC, g/L	163	516	3.8	3.3 – 19.4
FLLC, g/L	6	2.6	1.4	5.7 – 26.3
FKLC/FLLC ratio	27.2	200.8	2.8	0.26 – 1.65
Creatinine, µmol/L	131	741	119	55 – 96
Urea, mmol/L	7.4	26.1	9.7	2.5 – 7.5
Total protein, g/L	63.2	57.7	55	57.0 – 82.0
Calcium, mmol/L	2.36	3.42	2.31	2.08 – 2.65
Beta2-microglobulin, mg/L	2.85	69.9	5.06	1.09 – 2.53
LD, U/L	361	1051	603	208 – 378
NA – not available. FKLC – Free kappa light chains. FLLC – Free lambda light chains. LD – Lactate dehydrogenase. MCH – Mean cell haemoglobin. MCHC – Mean cell haemoglobin concentration. MCV – Mean cell volume.				

In October 2016, the patient was asymptomatic at the follow-up consultation. The concentration of FKLC increased to 289 mg/L, with a FKLC/FLLC ratio of 28.7. The SPE showed hypogammaglobulinemia of 4.2 g/L without monoclonal component. A slightly haemodiluted bone marrow aspiration analysis showed poor bone marrow reserve with an excess of plasma cells (5.5%). No bone marrow biopsy was performed due to the refusal of the patient. Plasma cells fluorescence *in-situ* hybridization (FISH) study demonstrated cytogenetic abnormalities including an immunoglobulin heavy locus (also known as IGH) rearrangement in 88% of the nuclei and three copies of cyclin-dependent kinases regulatory subunit 1 (CKS1B), a chromosome 1q marker, in 46% of the nuclei analysed. The patient was diagnosed with light-chain MM stage II according to the revised International Staging System for Myeloma classification (*17*). Due to the low production of monoclonal component and the absence of symptoms, a regular follow-up was scheduled without additional treatment.

**Figure 2:**
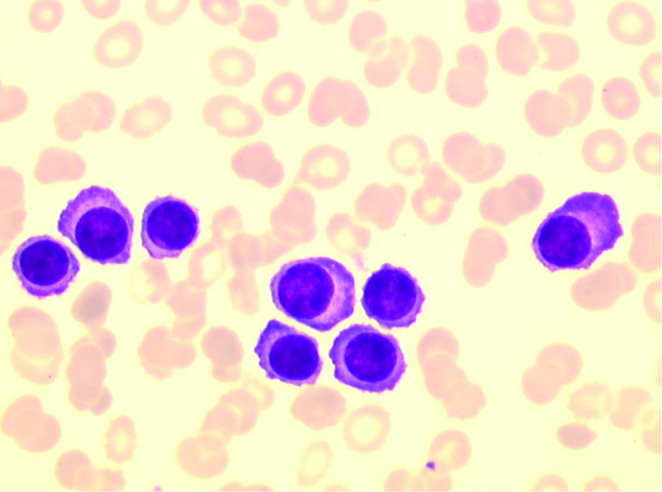
Blood smear from June 2018 showing the presence of circulating plasma cells.

**Figure 3:**
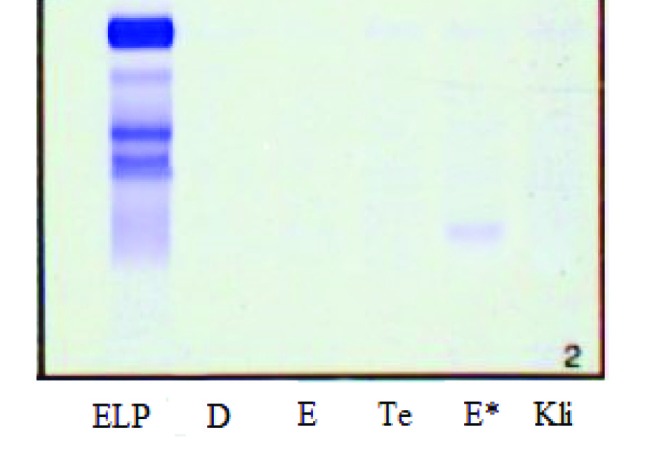
Immunofixation electrophoresis performed in June 2018 with a November 2015 frozen serum sample of the patient revealing a distinct slight band in IgE while using undiluted serum (E*). D - IgD migration profile; E* - IgE migration profile; ELP - reference profile of total protein electrophoretic migration; Kli - Kappa free light chain migration profile; Te - Negative control track.

In June 2018, the patient remained asymptomatic, but a systematic follow-up laboratory testing (Table 1) showed an acute renal failure with a glomerular filtration rate of 6 mL/min estimated by the CKD-EPI equation, hyperkalaemia of 6.1 mmol/L, hypercalcemia of 3.42 mmol/L, anaemia with haemoglobin of 101 g/L and extreme leucocytosis of 80.3 x10^9^/L. He was then admitted to intensive care for renal failure management. The blood smear performed with Sysmex SP1000-I (Sysmex Corporation, Kobe, Japan) showed 74% of plasma cells (Figure 2) which displayed a typical myeloma immunophenotype (CD38+, CD138+, CD28+, CD56+) analysed by flow cytometry Navios (Beckman Coulter, Fullerton, CA, USA). The SPE revealed for the first time a monoclonal peak in the gamma region, quantified at 3.4 g/L. The IFE using IgG, IgA, IgM, kappa and lambda antisera provided evidence of a slight monoclonal band for the kappa light chain. Further testing was carried out with IgE, IgD, kappa and free kappa-chain antisera. This second IFE exhibited a slight but distinct band for IgE and kappa light chain revealing for the first time the presence of an IgE-kappa monoclonal protein. Urinary immunofixation performed by Hydrasys 2 (Sebia) detected no Bence-Jones protein. A retrospective IFE was then performed using IgD, IgE, kappa and lambda antisera with the November 2015 patient’s frozen serum sample and revealed a slight distinct monoclonal band in the IgE (Figure 3). Interestingly, only testing pure serum, but not the 1:3 dilution recommended by the manufacturer, revealed the abnormal IFE pattern (*18*). The patient was diagnosed with IgE-producing plasma cells leukaemia secondary to an IgE-kappa MM. The patient had all the criteria of poor prognosis: PCL was secondary to MM, age ≥ 60 years, platelet count ≤ 100 x10^9^/L and peripheral blood plasma cell count ≥ 20 x10^9^/L (*19*). The decision was made to start treatment.

The patient received intravenous Pamidronate (60 mg/day) and adapted hydration to correct hypercalcemia. A renal replacement therapy resulted in a decrease of plasma potassium to 3.6 mmol/L, calcium to 1.95 mmol/L and creatinine to 342 µmol/L. As recommended, chemotherapy with the proteasome inhibitor bortezomib (1.3 mg/m^2^, twice a week) and dexamethasone (0.5 mg/kg, twice a week) was introduced (patient weight: 65 kg), decreasing the white-blood cell to 13.8 x10^9^/L after one week (*20*). The patient was discharged to the nephrology ward four days later to monitor the renal failure and to complete the first cycle of chemotherapy with bortezomib and dexamethasone.

A few days later, the patient was transferred to the haematology ward to receive the second and third chemotherapy cycles: bortezomib (1.3 mg/m^2^, twice a week), cyclophosphamide (300 mg/m^2^/week) and dexamethasone (40 mg/week); and the fourth chemotherapy cycle: bortezomib (1.3 mg/m^2^, twice a week), doxorubicin (36 mg/m^2^) and dexamethasone (40 mg/week). At the end of the four chemotherapy cycles in November 2018, laboratory results showed a leukocyte count of 3.1 x10^9^/L without circulating plasma cells. Serum protein electrophoresis showed complete disappearance of the monoclonal peak in the gamma-globulin region but IFE still detected the IgE-kappa monoclonal component. Free kappa light chains and FLLC were 3.8 and 1.4 mg/L, respectively, with a FKLC/FLLC ratio of 2.75. Due to an alteration of the general state and the development of peripheral neuropathy secondary to chemotherapy, the patient was admitted to a follow-up and rehabilitative care unit for palliative care and no further chemotherapy was performed. An informed consent form for the publication of a case report was signed by the patient during his last hospitalization in February 2019. He died in March 2019, 10 months after the diagnosis of sPCL.

## Discussion

This case report presents a very rare case of a 71-year-old male with IgE MGUS transforming into MM transforming to an IgE-producing sPCL. To our best knowledge, only 7 cases of IgE-producing sPCL have been described in the literature (*21*).

The detection and the identification of monoclonal immunoglobulin are cornerstones in the diagnosis of monoclonal gammopathy highlighting the role of the laboratory practitioner in the management of MM patients. The SPE in combination with serum FLC assay and IFE yields high sensitivity in the context of screening for MM and related plasma cell disorders (*14*, *15*). When a monoclonal peak consists only of light chains, an IFE specific for IgD and IgE is required (*5*, *22*, *23*). In our case, the recommended screening procedure to detect monoclonal gammopathies had been applied. The SPE and the IFE tests for monoclonal IgG, IgM, IgA and light chains kappa and lambda had not shown any abnormality that did not lead to suspect IgD and IgE gammopathies. In contrast, the increased serum FKLC and FLLC ratio supported the presence of a monoclonal gammopathy. Then, a faulty diagnosis of light chain MGUS was made in November 2015. To our knowledge, no guidelines specify whether an abnormal FLC test should lead to the detection of IgD and IgE monoclonal gammopathy in the absence of an isolated light chain (*22*). In our case, an IgD and IgE IFE could have rectified the diagnosis to an IgE-Kappa MGUS as early as 2015. Interestingly, the 1:3 serum dilutions for IFE recommended by the manufacturer did not detect the production of IgE monoclonal immunoglobulin while IFE performed without prior dilution showed clear bands in IgE. Thus, the 1:3 dilution seems to decrease the sensitivity of the IFE compared to the non-diluted test. On the one hand, the manufacturer indicates that this dilution reduces the risk of non-specific fixation, but on the other hand, the use of a control track without anti-serum allows the presence of these non-specific fixations to be detected. A similar report was made by Bossuyt *et al.*, who observed a case of light chain disease missed by IFE with Hydrasys when the sample was diluted 1:3, but not when the sample was analysed undiluted (*23*).

Serum protein electrophoresis quality controls were performed before each series with the Sebia normal control serum (PN 4785) and after each series with the Sebia hypergamma control serum (PN 4787). Quality controls for IFE (IT/IF control PN 4788) were performed once a week. No quality control of IgD and IgE immunofixation has been performed. No standard has been used. Dilutions of sera before immunofixation were 1:6 for IgG and 1:3 for IgA, IgM; IgD, IgE, FKLC and FLLC excepted with the 2015 sample performed retrospectively in June 2018 without diluting IgD and IgE.

The absence of urinary immunofixation at the time of screening followed the recommendations of the International Myeloma Working Group: “In the context of screening, the serum FLC assay in combination with serum protein electrophoresis and immunofixation yields high sensitivity, and negates the need for 24h urine studies for diagnoses other than light chain amyloidosis” (*14*). To our knowledge, no guidelines require urinary immunofixation in the presence of a single FLC abnormality. According to Kyle RA and Rajkumar, the search for Bence-Jones proteinuria is only necessary during follow-up (*15*). Nevertheless, it appears in retrospect that it would have been useful to perform urinary immunofixation at the time of diagnosis.

French legislation requires that samples be stored frozen as soon as tumour markers have been tested for further analysis if necessary. In this case, the sample used for the measurement of β2-microglobulin in 2015 was stored at - 20°C and used in 2018 to perform the IFE. The literature on the storage stability of immunoglobulins is quite poor and no data are available to our knowledge on IgE stability. Glislefoss *et al.* did not observe any significant difference in IgG measurement before and after 25 years of storage at - 25°C (*24*). However, this alone cannot guarantee that immunofixation as a whole has retained all the initial characteristics 3 years later.

In a recent literature review of 63 IgE MM cases, Hejl *et al.* observed that IgE MM has a more aggressive clinical course than others MM subtypes (*5*). However, IgE monoclonal gammopathies do not necessarily imply malignant evolution and benign courses are indeed possible (*5*, *25*, *26*). The IgE MM patients mean age at the time of diagnosis was 68 years (range: 28 – 87 years) with a male predominance (sex ratio M/F: 1.26). Approximately 45% of patients had anaemia, 27% had renal failure, 17% had hypercalcemia and β2-microglobulin was > 3 mg/L in 63% of cases. The IgE migrated predominantly in the gamma region and were associated with a kappa light chain in 63% of cases. Bone lesions were present in 56% of cases. The authors pointed out that the characteristics of IgE MM are very similar to those of other MM types with the exception of a poorer prognosis. The patient described herein had similar epidemiological characteristics but had no anaemia, no hypercalcemia, and no bone lesions at the time of MM diagnosis.

Secondary PCL is a rare late-stage complication of relapsed/refractory myeloma (*27*). Patients with sPCL were generally about 60 years old and they frequently had anaemia, thrombocytopenia and bone lesions (*28*). Multiple myeloma cells and sPCL cells are known to secrete inflammatory cytokines like tumour necrosis factor alpha, interleukin-1 beta and interleukin-6 that enhance osteoclast proliferation and activity while inhibiting osteoblast bone formation (*29*). In addition, the expansion of sPCL cells deregulates the bone compartment. These explained the patient’s hypercalcemia at the time of sPCL diagnosis resulting in acute renal failure due to extracellular dehydration. The patient developed IgE-producing sPCL 31 months after the initial diagnosis of MGUS. This corresponds to the median time reported by Jurczyszyn *et al.* to develop sPCL from MM (*30*). A strong association between IgE MM and sPCL has been suggested by Hejl *et al.* in his review: 7 of the 63 IgE MM patients reported have developed a sPCL (*5*). However, this statement should be viewed with caution. On the one hand, the review was not a systematic review of the literature, and on the other hand, drawing epidemiological conclusions from case series necessarily expose the reader to the risk of publication bias. A systematic study of the IgE MM patient population would be necessary to obtain more reliable data.

Although Avet-Loiseau *et al.* reported the translocation t (*11*, *14*) as the hallmark feature of IgE myeloma and t (*11*, *14*)(q13;q32) was associated with PCL, these cytogenetic abnormalities were not observed at either the MM or sPCL stages in the case of our patient (*31*).

The International Myeloma Working Group provides recommendations for primary PCL treatment (*26*).To our knowledge, there is no standardized recommendation for the management of sPCL. Induction therapy needs to be promptly initiated and has high clinical activity leading to rapid disease control in an effort to minimize the risk of early death (*32*). A recent study showed an improvement in prognosis through treatment with bortezomib-containing regimens which was the treatment chosen for the patient (*11*). As in MM, treatments with thalidomide and lenalidomide are likely to have some effect on sPCL (*33*). The survival is still poor, and few patients achieve remission for more than 1 year.

Several limitations to the conclusions of this case report must be considered. This is a rare case not chosen from representative population samples, so we cannot generate information on rates, incidences or prevalence. This case report could strengthen the hypothesis that the IgE myeloma subtype may expose the patient to an increased risk of developing aggressive leukaemia compared to other subtypes but this generalization must be taken with caution.

## Conclusion

IgE myeloma is the rarest subtype of myeloma and it has probably the worst prognosis. The medical laboratory plays an important role in the proper diagnosis and quality of management of MM patients. This case revealed that an abnormal FLC ratio should be considered as a warning signal suggesting the possibility of IgD or IgE myeloma even in the absence of a solitary light chain in IFE. In addition, the use of an undiluted serum could increase the sensitivity of the immunofixation for the detection of IgE and IgD monoclonal gammopathies compared to the 1:3 dilution recommended by the manufacturer. Further investigation is warranted to evaluate the diagnostic strategy of systematically testing IgD and IgE with IFE in the presence of an abnormal FLC ratio.
